# Conflict at Work Impairs Physiological Recovery during Sleep: A Daily Diary Study

**DOI:** 10.3390/ijerph191811457

**Published:** 2022-09-12

**Authors:** Katja Kerman, Roman Prem, Bettina Kubicek, Edo Meyer, Sara Tement, Christian Korunka

**Affiliations:** 1 Department of Psychology, Faculty of Arts, University of Maribor, 2000 Maribor, Slovenia; 2 Institute of Psychology, University of Graz, 8010 Graz, Austria; 3 Department of Applied Psychology: Work, Education and Economy, Faculty of Psychology, University of Vienna, 1010 Wien, Austria

**Keywords:** work stress, sleep, heart rate variability, RMSSD, HF, perseverative cognition

## Abstract

Sleep plays an essential role in maintaining employees’ health and well-being. However, stressors, such as conflict at work, may interfere with employees’ sleep. Drawing on previous literature on the relationship between conflict at work and sleep outcomes, we proposed a negative relationship between daily conflict at work and physiological changes during early sleep, particularly nocturnal heart rate variability (HRV). Furthermore, building on the perseverative cognition hypothesis, we proposed that daily work-related rumination mediates the relationship between conflict at work and nocturnal HRV. Ninety-three healthcare employees participated in a daily diary study for five workdays, resulting in 419 observations. Multilevel analysis revealed a significant relationship between daily conflict at work and nocturnal HRV, specifically high-frequency (HF) power. Daily conflict at work was found to predict rumination; however, rumination did not significantly predict nocturnal HRV. Our results suggest that daily conflict at work increases rumination during the off-job time and may directly alter nocturnal HRV, specifically parasympathetic function in early sleep.

## 1. Introduction

Healthy sleep is essential for maintaining employees’ health and well-being [1]. However, studies suggest that work-related stress is one of the most common factors contributing to sleep disturbances [2] and poor sleep quality [3,4,5]. Although most studies examining the relationship between work stress and sleep have relied on self-reported measures of sleep quality or sleep disturbances (e.g., [5,6,7]), sleep physiology can also be altered by work-related stressors. Specifically, early sleep can be altered by secretory activity of the hypothalamic-pituitary-adrenal (HPA) axis [8]. The HPA axis represents the physiological stress response system, and its activation results in physiological stress response in which the autonomic nervous system (the sympathomedullary pathway) and the endocrine system release stress-related neurotransmitters and hormones, such as catecholamines (norepinephrine, epinephrine) and cortisol [9]. During early sleep, which is predominated by slow-wave sleep, the HPA axis activity is down-regulated to a minimum, accompanied by a decrease in the release of adrenocorticotropin and cortisol. In other words, the nadir activity of the HPA axis in early sleep reflects an active inhibition of the stress response system. However, daily stressors may stimulate the secretion of cortisol, which could attenuate the inhibition of the HPA axis during early sleep [8]. Indeed, a study by Hall et al. [10] found a relationship between acute stress and nocturnal HRV that showed decreased parasympathetic activity during NREM sleep, with parasympathetic modulation slowly increasing during each NREM phase. Workplace conflict is considered a severe work-related stressor that can vary in intensity (minor disagreement to aggressive behavior) and can be overt (e.g., rudeness) or covert (e.g., spreading false rumors [11]). The prevalence of conflict and incivility in the workplace is estimated to be approximately 71% of employees in the public service sector [12] and, according to a meta-analysis, ranges from 67.5% to 90.4% in nurses [13]. Although previous studies have found that workplace conflict is associated with poor employee health and well-being [14,15], there is a lack of studies examining how conflict at work relates to employees’ restful sleep. The literature on workplace incivility, a construct related to conflict at work (see [16]), offers some insights, showing that incivility at work predicts sleep-related outcomes, such as insomnia [17,18]. However, there is a gap in understanding whether sleep physiology, specifically nocturnal HRV, can also be altered due to conflict at work. In addition to the direct effects of work stressors on sleep physiology, work-related perseverative cognition (e.g., rumination) may serve as an additional pathway linking conflict at work and nocturnal HRV. Perseverative cognition is a reaction to common daily stressors, such as stressful work situations [19], that prolongs the stress response because the stressor remains cognitively present even after exposure to the stressor has ceased [20]. Thus, perseverative cognition not only prolongs employees’ cognitive exposure to work-related stressors but also prolongs the stress response, providing an additional pathway to sleep and other health-related outcomes [9,20]. Although previous studies demonstrate the mediating role of work-related rumination in the relationship between workplace conflict and insomnia [17], it remains largely unexamined whether the mediating role can also be found when examining physiological changes in sleep. To the best of our knowledge, there is a lack of studies examining whether conflict at work may be detrimental to healthy sleep. Based on the literature gaps, the present study examined the relationship between daily conflict at work and nocturnal heart rate variability during early sleep, as well as investigated the mediating role of work-related rumination during off-job time.

## 2. Materials and Methods

### 2.1. Design and Procedure

To assess daily variability in conflict at work, work-related perseverative cognition, and nocturnal HRV, we utilized a daily diary study design. Conflict at work was measured after work, and work-related rumination and control variables were assessed before sleep. HRV was recorded during sleep using a small, portable electrocardiogram (ECG) heart rate monitor. The eMotion Faros 180° is certified as a medical device (EN60601-1; eMotion Faros, Mega Electronics Ltd, Finland) and uses two electrodes to capture the ECG signal (with a resolution of 1000 Hz). The use of this device follows recommendations on HRV data collection, as it enables the researcher to visually inspect the QRS complex and directly obtain HRV indicators [21]. We collected data from a sample of employees working in elderly health care homes because they frequently face conflict and incivility at work [13]. Participants were informed about the study and given instructions on using the ECG devices and daily diaries. Because most of the employees working in health care (e.g., nurses) perform shift work, they were instructed to participate for five workdays and only on days when they worked day shifts (within the time frame of two weeks). Because it is unclear whether sleep measurements after a day shift are comparable to measurements after a night shift due to factors such as circadian rhythm [21,22,23], this design provided a relatively standardized measurement to explore within-person effects. Thus, most of the sample (92.55%) participated on nonconsecutive workdays, with an average of 2.3 days (*SD* = 1.8) between measurements.

### 2.2. Participants

Ninety-three employees participated in the study for an average of 4.5 days, resulting in 419 observations. The majority of the sample was female (78.3%), with a mean age of 41.64 years (*SD* = 10.7). A large proportion of our sample (74.6%) worked as healthcare providers, and 25.4% were administrative or other staff. Nearly half of the participants (42.7%) had completed vocational school and 22.4% of participants had completed an apprenticeship. The tenure of the sample was 10.8 years (*SD* = 8.8), and 39.2% of participants occupied a leadership position. On average, participants worked 39.6 h per week (*SD* = 5.6), and 41% worked shifts.

### 2.3. Self-Report Diary Measures

***Interpersonal Conflict at work***. The Interpersonal Conflict at Work Scale [11] was adapted for daily measurement of conflict at work (sample item: "How often did you get into arguments with others at work today?”). Items were assessed on a five-point scale ranging from *Not at all* to *Very often (several times per hour)*. The scale has adequate internal consistency on the within-person (α = 0.7) and the between-person level (α = 0.8). ***Work-related rumination***. To measure daily work-related rumination, the Negative Rumination subscale from the Negative and Positive Work Rumination Scale [24] was adapted for the daily measurement. The four items (sample item: “Today after work, did you keep thinking about the negative things that happened at work?’’) were assessed on a five-point scale, ranging from *Not at all* to *To a great extent* (within-person and between-person level α = 0.8 and 0.9, respectively ).

### 2.4. Control Variables and Exclusion Criteria

Per recommendations (see [21]), we excluded participants with chronic heart disease and those taking cardioactive medications. Participants were asked to report their daily nicotine, alcohol, and caffeine consumption and medication use. Daily physical activity was measured using the Saltin–Grimby Physical Activity Level Scale [25], which measures activity level on a four-point scale, ranging from *Sedentary* to *Hard physical training for competitive sports*. Age and sex were included as between-person level control variables. Finally, in line with studies finding that anxiety and depression may impact both HRV and sleep [26,27,28], the depression (α = 0.86) and anxiety (α = 0.65) subscales from the Depression Anxiety Stress Scale [29] were also included as control variables on the between-person level.

### 2.5. Heart Rate Variability Assessment and Analysis

In our analysis of nocturnal HRV, we focused on the first 90 min of sleep, which enabled us to capture the first sleep cycle [30]. This decision was made based on previous research showing that slow-wave sleep, which dominates the first sleep period [30,31], provides a highly standardized condition for measuring HRV [32] and research indicating that acute stress may be more accurately reflected in the early stages of sleep [8,33]. We focused on the root mean square of successive differences (RMSSD) (RMSSD is obtained by squaring each time difference between successive heart beats and then calculating the square root of the averaged difference [34]) and the high-frequency band (HF) (obtained by normalizing the power of the high-frequency band [34]) as their physiological origin comes from the vagal tone and because previous studies show high reproducibility of both indicators during NREM sleep [32]. HRV analysis was performed using Kubios Version 3.0 software [34]. First, each recording was visually inspected to examine its quality and validate the R-peaks detected by the algorithm. To ensure the quality of the recordings, we excluded recordings with signal losses or containing more than 1% ectopic heartbeats (2.14% of the recordings were excluded). Considering that measurements outside of a laboratory setting almost always contain artifacts, such as movement or missed beats [35,36], and that such artifacts reduce the reliability of HRV analysis [36], artifacts were replaced by using interpolated RR values [34]. The average percentage of corrected beats per recording was 0.34% (*SD* = 0.31). The 90-min interval was then divided into 18 five-minute epochs for analysis [21]. Finally, RMSSD and HF were extracted for each epoch, and the average value of RMSSD and HF across all five-minute epochs was calculated.

### 2.6. Statistical Analyses

Because of the hierarchical nature of our data (observations are nested within individuals), we took a multilevel approach to the analysis. The multilevel approach decomposes the variance into between- and within-group components and thus allows for different relationships on the within- and between-person level [37]. First, we inspected the interclass correlations (ICC). The ICC values for conflict at work (ICC = 0.47), rumination (ICC = 0.62), RMSSD (ICC = 0.82), and HF (ICC = 0.83) confirmed that a multilevel approach was justified. We tested the hypothesized direct effects in a multilevel model in Mplus 8, both at the within-person (level 1) and the between-person (level 2) levels. All relevant within- and between-person level control variables were included in the initial model. However, in light of model parsimony, all nonsignificant control variables were excluded from the final model (see [38]), leaving physical activity predicting RMSSD and nicotine intake predicting HF on the within-person level. Age and sex were were included as control variables on the between-person level for both RMSSD and HF, and depression was included as a predictor of HF. Correlation terms between HF and RMSSD were included in the model.

## 3. Results

Fit indices showed adequate model fit (AIC = 5037.853 χ^2^ = 19.11, *df* = 8, RMSEA = 0.06, CFI = 0.91, TLI = 0.71). The means, standard deviations, and correlations between the variables of interest are shown in Table 1.

**Figure 1:**
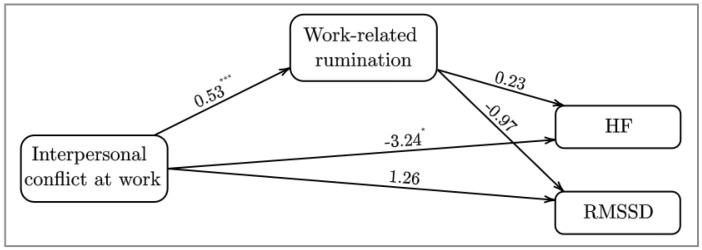
Unstandardized estimates and *p*−values of day−level estimates of the direct effects. * *p* ≤ 0.05. *** *p* ≤ 0.001.

The unstandardized results of the within-person level model examination are depicted in Figure 1. The results showed that daily interpersonal conflict at work significantly and positively predicted daily work-related rumination. However, rumination did not significantly predict either RMSSD or HF. Furthermore, daily interpersonal conflict at work significantly and negatively predicted HF but did not significantly predict RMSSD.

The standardized and unstandardized results at the between-person level can be found in Table 2. The results on the between-person level showed that conflict at work had a significant and negative effect on HF, but not RMSSD, and a significant and positive effect on rumination. Work-related rumination did not significantly predict nocturnal RMSSD or HF. Thus, the between-person level results are largely consistent with the within-person level results. Because of the nonsignificant b-paths at the within- and between-person levels, we did not calculate the indirect effects.

## 4. Discussion

In the present study, we investigated the relationship between interpersonal conflict at work, work-related perseverative cognition (specifically rumination), and nocturnal HRV. By relying on objectively measured physiological changes during sleep, namely RMSSD and HF, as parameters of HRV, this study overcomes the methodological shortcomings of previous literature. Although previous studies provide valuable insights into the relationship between conflict at work and healthy sleep [17,18,39], they mostly rely on self-reported sleep outcomes. Therefore, it is unclear whether conflict at work may also be linked to physiological changes in early sleep. In addition, the present study utilized a daily diary design, which allowed us to examine the proposed model both at the within- and between-person level. Results showed that daily interpersonal conflict at work negatively predicted HF, suggesting that conflict at work is related to altered nocturnal HRV, particularly parasympathetic function in early sleep. In other words, on days when employees experience conflict at work, it is accompanied by physiological changes during sleep. Additionally, the between-person results indicate differences in employees, i.e., those who experience more conflict at work also show decreased parasympathetic activity in early sleep. Our findings are consistent with previous studies showing a negative association between conflict at work and employee sleep [17,18] and the negative effects of acute stress on parasympathetic modulation during sleep [10]. Notably, our findings are in line with the notion that acute stress can impair restorative sleep via blunted parasympathetic activity [10]. Although examining the occurrence of insomnia is beyond the scope of the present study, our results also suggest that work stress, particularly conflict at work, may lead to hyperarousal during early sleep, possibly contributing to the development of insomnia (see [40,41]). On the other hand, daily conflict at work did not significantly predict RMSSD. Although both RMSSD and HF indicators reflect cardiac vagal control, some studies argue that RMSSD is not a pure measure of cardiac vagal control but may also reflect sympathetic influences [42]. On the other hand, parasympathetic activity has been shown to be a major contributor to the HF component of HRV in clinical studies [43]. This is especially true when the respiratory rate is between 9 and 24 cycles per minute, reflective of respiratory sinus arrhythmia [21]. Indeed studies show that early sleep (especially slow-wave sleep) is an optimal condition for measuring respiratory sinus arrhythmia, or in other words, for detecting vagal influences on the HRV via HF [44]. Beyond the direct relationship between daily conflict at work and nocturnal HRV, we also examined the mediating role of daily work-related perseverative cognition, specifically rumination. Consistent with previous studies [17,45], our results showed that conflict at work positively predicted work-related rumination; that is, the more conflict at work employees experience on a given day, the more likely they are to have negative ruminative thoughts about work during the non-work time (on the same day). This relationship was also statistically significant on the between-person level, i.e., employees who experience more conflict at work also experience more frequent work-related rumination after work. However, in contrast to the perseverative cognition hypothesis [20], our results do not lend support to the mediating role of daily rumination. Specifically, daily work-related rumination did not significantly predict nocturnal HRV. It is possible that the nonsignificant effects are due to the operationalization of work-related perseverative cognition in our study. We examined rumination, which captures the past-oriented aspect (e.g., thinking back to negative work events), whereas the perseverative cognition hypothesis proposes the distinction between past-oriented (i.e., rumination) and future-oriented (i.e., worry) perseverative thoughts [20]. Thus, it is possible that worry is more related to prolonged stress response or anticipatory stress than rumination. Second, we only captured the frequency but not the duration of work-related ruminative thoughts. For example, Brosschot et al. [46] examined both frequency and duration of worry in predicting nocturnal HRV and found that the duration of worry is more robust in predicting nocturnal HRV than the frequency. Although similar research on work-related rumination is scarce, it may be feasible that not only the frequency but also the duration of work-related rumination plays a relevant role in nocturnal HRV. The present study is not without limitations. Our study focused on rumination and captured the past-oriented aspect of perseverative cognition. Future studies might benefit from measuring both forms of perseverative cognition while also capturing past-oriented (rumination) and future-oriented (worry) aspects. Furthermore, our study measured the frequency of rumination but not the duration. However, a study by Brosschot et al. [46] found that the duration of perseverative thought may also predict nocturnal HRV. Thus, information on the frequency and duration of work-related perseverative thoughts could provide additional insight into the relationship between conflict at work, perseverative cognition, and nocturnal HRV. In addition, our sample combines shift and non-shift employees. Although all measurements were taken only after day shifts, the between-person results should be interpreted cautiously, as studies find that sleep outcomes may differ between shift- and non-shift workers [23]. Finally, to minimize the burden on the participants, we did not conduct a morning measurement in our study, which prevented us from measuring self-reported sleep quality. It would be interesting to investigate in future research whether physiological changes during sleep due to conflict at work correspond with employees’ self-reported sleep quality.

## 5. Conclusions

Our findings provide valuable insights into the interplay between daily conflict at work, rumination, and nocturnal HRV. Daily conflict at work was found to predict rumination after work and nocturnal HRV, highlighting the potentially detrimental consequences for employees’ off-job recovery and sleep. Given that stress-related changes in nocturnal HRV may adversely affect employees’ sleep, health, and well-being [10], organizations should pay close attention to conflict and incivility. Studies show that the negative effects of conflict at work can be mitigated by organizational support [47] and co-worker support [48]. Therefore, organizations, especially healthcare institutions, should strive to improve organizational and social support to protect their employees’ healthy and restorative sleep. Lastly, conflict at work can be decreased to a great extent by a fair distribution of workload and securing organizational justice and fairness [49].

## Figures and Tables

**Table 1:** Means, standard deviations, and standardized zero-order correlations.

	1	2	3	4	5	6	7	8	9
1. Conflict at work		0.55 ***	−0.05	0.04			−0.22 *	0.05	0.05
2. Rumination	0.28 ***		−0.03	−0.06			−0.04	0.09	0.33 ***
3. HF	−0.09	−0.01		0.50 ***			−0.52 ***	0.31 ***	−0.18
4. RMSSD	0.01	−0.04	0.28 ***				−0.55 ***	0.01	−0.11
5. Physical activity	0.04	0.01	−0.01	−0.14 *					
6. Nicotine intake	0.04	0.14	−0.14	−0.32 ***	−0.11 *				
7. Age								0.07	-0.09
8. Sex									0.01
9. Depression									

Note. Within-person level correlations are displayed below the diagonal, and between-person level correlations are displayed above the diagonal. CAW = conflict at work. HF = high frequency (normalized units). RMSSD = root mean square of successive differences (normalized units).* *p* ≤ 0.05. *** *p* ≤ 0.001.

**Table 2:** Standardized and unstandardized within- and between-person level estimates.

	Est.	S.E.	Est./S.E.	*p*	Std. Est.
Within-person level estimates					
CAW → Rumination	0.53	0.17	3.11	<0.01	0.26
CAW → RMSSD	1.28	2.75	0.47	0.64	0.03
CAW → HF	−3.30	1.55	−2.13	0.03	−0.14
Rumination → RMSSD	−0.97	1.30	−0.74	0.46	−0.05
Rumination → HF	0.23	0.72	0.31	0.75	0.02
Physical activity → RMSSD	−6.10	2.12	−2.88	<0.01	−0.15
Nicotine intake → HF	−0.71	0.37	−1.90	0.06	−0.15
RMSSD ↔ HF	15.18	5.18	2.93	<0.01	0.21
Between-person level estimates					
CAW → Rumination	1.26	0.28	4.60	<0.01	0.45
CAW → RMSSD	−0.70	8.44	−0.08	0.93	−0.01
CAW → HF	−13.19	4.83	−2.73	<0.01	−0.25
Rumination → RMSSD	−2.19	3.27	−0.67	0.50	−0.07
Rumination → HF	2.78	2.06	1.35	0.18	0.15
Age → RMSSD	−1.24	0.24	−5.26	<0.01	−0.58
Sex → RMSSD	5.08	7.92	0.64	0.52	0.08
Age → HF	−0.83	0.13	−6.33	<0.01	−0.63
Sex → HF	14.94	3.46	4.32	<0.01	0.38
Depression → HF	−9.31	2.78	−3.35	<0.01	−0.24
RMSSD ↔ HF	64.11	24.21	2.65	<0.01	0.34

Note. Est. = estimate. S.E. = standard error. Std. Est. = standardized estimate. RMSSD = root mean square of successive differences (normalized units). CAW = conflict at work. HF = high frequency (normalized units). Single-headed arrow indicates a regression term, and a double-headed arrow indicates a correlation term.

## Data Availability

The data that support the findings of this study are available from the corresponding author upon reasonable request.
